# Treatment Compliance of Multidrug Resistant Tuberculosis in Uzbekistan: Does Practice Follow Policy?

**DOI:** 10.3390/ijerph18084071

**Published:** 2021-04-12

**Authors:** Ruzilya Usmanova, Nargiza Parpieva, Hayk Davtyan, Olga Denisiuk, Jamshid Gadoev, Sevak Alaverdyan, Kostyantyn Dumchev, Irina Liverko, Barno Abdusamatova

**Affiliations:** 1 Republican Specialized Scientific-Practical Medical Center of Phthisiology and Pulmonology of the Republic of Uzbekistan, 1 Majlisiy Street, Shayhantahur District, Tashkent 100086, Uzbekistan; nargizaparpieva@gmail.com (N.P.); liverko@yandex.ru (I.L.); 2 Tuberculosis Research and Prevention Center, NGO, 38 Apartment, 33 Charents Street, Nor Hachn 2412, Armenia; haykdav@gmail.com; 3 Alliance for Public Health, Building 3, 24 Bulvarno-Kudryavska Street, 01601 Kyiv, Ukraine; o.denisiuk@gmail.com; 4 World Health Organization (WHO) Country Office in Uzbekistan, 16 Tarobiy Street, Tashkent 100100, Uzbekistan; gadoevj@who.int; 5 Bielefeld Graduate School of Economics and Management (BiGSEM), Bielefeld University, 25 Universitätsstraße, 33615 Bielefeld, Germany; s.alaverdyan@iset.ge; 6 Ukrainian Institute of Public Health Policy, Biloruska Street, 5, 02000 Kyiv, Ukraine; dumchev@uiphp.org.ua; 7 Department of Protection of Maternity and Childhood of the Ministry of Health, 12 Navoi Street, Shayhantahur District, Tashkent 100011, Uzbekistan; barno.abdusamatova@minzdrav.uz

**Keywords:** MDR/RR TB, weight-dosage compliance, national guideline compliance, operational research, SORT IT

## Abstract

Compliance with treatment guidelines is essential to achieve successful outcomes in tuberculosis patients. Thus, we assessed if multidrug-resistant tuberculosis treatment practices from 2012–2018 in Uzbekistan were compliant with national guidelines in terms of regimens prescribed, weight-based drug dosages used, and documentation of treatment changes (such as prolongation of intensive phase, change of drugs, and their reasons) in the treatment card and Consilium form. A total of 1481 patients were included. Of them, only 25% received standardized regimens as per guidelines and the remaining received individualized regimens. There was an increasing trend in using standardized regimens from 2% in 2012 to 44% in 2018. Compliance to recommended weight-based drug dosages was observed in 85% of the patients during the intensive phase and 84% in the continuation phase—ranged 71–91% over the years. Prolongation of the intensive phase was done in 42% of patients. The treatment was changed in 44% of patients during the intensive phase and 34% of patients during the continuation phase. The documentation of treatment changes was suboptimal (42–75%) during the initial years (2012–2014); however, it improved significantly during later years (86–100%). Future research should explore reasons for non-compliance so that the quality of patient care can be improved.

## 1. Introduction

Tuberculosis (TB) is one of the top ten causes of death worldwide. Among all infectious diseases, TB accounts for the highest number of deaths. According to the World Health Organization (WHO) estimates, 10 million people developed TB in 2019, and 1.2 million people died due to TB [1].

Globally, the TB incidence rate is decreasing on average by 2% per year. In order to reach the 2025 milestone set by the End TB strategy (World Health Organization), it would be necessary to increase the rate of decline to 10% annually [2]. There is a substantial variation in the TB incidence rate in different countries, and this may be related to socioeconomic status. In all high-income countries, the incidence rate of new TB cases is lower than 10 per 100,000 population. On the other hand, in countries with lower income levels, incidence rates can be 500 or more per 100,000 [1].

Drug-resistant (DR) TB is another dangerous public health challenge. The estimated number of rifampicin-resistant (RR) TB patients in 2019 was 465,000 (95% Confidence Interval (CI) 400,000–535,000). Of these, 78% had multidrug-resistant (MDR) TB, with resistance to both rifampicin and isoniazid. The proportion of MDR/RR-TB patients was 3.3% among new patients and 18% among the previously treated patients. However, in the countries of the former Soviet Union, the burden of MDR/RR-TB is higher, and the percentage among previously treated patients can be 50% or higher [1]. WHO recommends different regimens for the treatment of MDR/RR-TB lasting from 9–24 months [3]. Among patients who started treatment for MDR/RR-TB in 2017, only 57% had a successful treatment outcome, and 15% died during the course of the treatment [1].

In Uzbekistan, the WHO estimated that the number of incident cases (new and relapse) was 22,000 (95% CI 15,000–30,000) in 2019, while in the same year number of estimated MDR/RR TB cases was 3200 (95% CI 2200–4400). The proportion of MDR/RR-TB among new and previously treated patients was 12% (95% CI 11–13%) and 22% (95% CI 20–24%), respectively [1]. National guidelines for programmatic management of DR-TB in Uzbekistan were developed in collaboration with WHO experts and approved by the Ministry of Health of the Republic of Uzbekistan in 2014 [4,5,6]. The treatment success rate among MDR/RR-TB patients who started treatment in 2017 was reported to be 61% [1]. Compliance with the treatment regimens that contain weight-based dosing is essential for achieving good treatment outcomes [7,8].

There has been no formal evaluation of whether treatment practices are compliant with the recommendations in the orders and national guidelines on DR-TB. Hence, we conducted this study to assess whether MDR-TB treatment practices in Uzbekistan from 2012–2018 were compliant with the national treatment guidelines in terms of: (i) correct prescription of initial standardized treatment regimens; (ii) correct weight-based drug dosages; and (iii) correct documentation of treatment changes (prolongation of intensive phase and regimen changes during both phases of treatment). Additionally, we assessed factors associated with compliance with weight-based dosing.

## 2. Materials and Methods

### 2.1. Study Design

We conducted a cohort study using secondary data obtained under the pilot project titled “Evaluation of the TB drug usage for treating MDR TB” from 2012–2018 by the National TB Program (NTP) of Uzbekistan.

### 2.2. Setting

#### 2.2.1. General Setting

Uzbekistan is located in Central Asia and has a population of 34 million. It is divided into twelve regions, one autonomous republic (the Republic of Karakalpakstan) and the Tashkent metropolitan area; the capital city [9].

#### 2.2.2. Specific Setting

The NTP of the Republic of Uzbekistan coordinates and oversees all TB control activities in the country. Implementation of the program is carried out by the Republican Specialized Scientific-Practical Medical Center of Phthisiology and Pulmonology (RSSPMCPP). TB care is provided free of charge to all patients (including DR-TB patients) throughout the country. All patients receive treatment in accordance with the national guidelines, which align with WHO-recommended treatment strategies. Regional specialized dispensaries/hospitals and specialized TB units located in primary health care (PHC) facilities provide care to TB patients. Treatment is prescribed by TB specialists at the start of the treatment in the inpatient units. Individual-level data are collected routinely at all levels of care in accordance with WHO definitions and reported upstream to the NTP. During the study period, the diagnosis of DR-TB was made using phenotypic culture and drug susceptibility tests, line probe assay (HAIN), and Xpert MTB/RIF assay. Sputum samples were collected at the provincial dispensaries and transported to the nearest laboratory for testing. Treatment was started based on the results of rapid tests, and adjustments were made after culture, and drug susceptibility testing results were available. For every diagnosed DR-TB patient, the treating doctor presents the case (the clinical profile and drug resistance status) at the weekly medical Consilium of TB specialists held at the provincial level. The medical Consilium recommends the treatment regimen to be used in accordance with national guidelines. Any changes to the treatment regimen (such as when additional resistance is detected) or prolongation of treatment have to be discussed and approved by the medical Consilium, except temporary changes in treatment done to treat adverse drug reactions. During the study period, the Global Fund to Fight AIDS, Tuberculosis and Malaria (GFATM) supplied the drugs for treatment, except in the Republic of Karakalpakstan, where Médecins Sans Frontières managed the drug supply.

Drug-susceptible and DR-TB patients are treated in accordance with the 2014 Order of the Ministry of Health of the Republic of Uzbekistan, in line with the WHO guidelines [4]. Before 2014, treatment of MDR-TB patients consisted of three standardized regimens, and the duration of treatment was 24 months: six months of intensive phase (with injectables) and 18 months of continuation phase (without injectables) [6]. In 2014, the “National Guidelines for the Program Management of Drug-Resistant Tuberculosis of the Republic of Uzbekistan” was adopted, which recommended an MDR-TB treatment regimen with an intensive phase duration of 8 months [5]. In the 2014 order, individualized regimens tailored to the drug susceptibility pattern of the patients were also approved [4].

#### 2.2.3. “Evaluation of the TB Drug Usage for Treating MDR TB” Project

The project aimed to monitor the correct usage of the drugs for MDR TB throughout the country. Experts from the national level visited treatment facilities and collected information with regards to the available drug stocks, and they also reviewed Consilium forms and patient cards. All collected data was captured in a dedicated project database which was different from the routinely collected programmatic data.

### 2.3. Study Population

All MDR/RR-TB patients who received second-line TB treatment in Uzbekistan from 2012–2018 and assessed under the project “Evaluation of the TB drug usage for treating MDR TB” were included.

### 2.4. Data Variables, Sources of Data, and Data Collection Instrument

Data variables included patient identification number; date of starting treatment, treatment regimen, region, age, sex, weight at the start of intensive phase and continuation phase, dosage compliance in intensive and continuation phase, prolongation of intensive phase, treatment changes in intensive and continuation phase and their correct documentation in patient cards and Consilium forms.

Operational definitions: The treatment regimen was considered ‘standardized’ if the initial treatment regimen aligned with the national guidelines. The dosage was assessed to be compliant if all the medications used during the treatment (calculated separately for both treatment phases and for the whole treatment) were the same as in the weight-based charts of the national guidelines. If the reason for the treatment change (any) was recorded in the patient card and in Consilium form, then it was regarded as “correct documentation”.

The Source of data was the “Evaluation of the TB drug usage for treating MDR TB” project database.

### 2.5. Analysis and Statistics

Proportions were used to summarize categorical variables. Means and standard deviations were used to summarize continuous variables. Risk factors associated with the non-compliance with the drug dosage were analyzed using log-binomial regression, and adjusted risk ratios (aRR) with 95% confidence intervals (CI) were calculated. Levels of significance were set at 5% (*p*-value < 0.05).

## 3. Results

**Figure 1:**
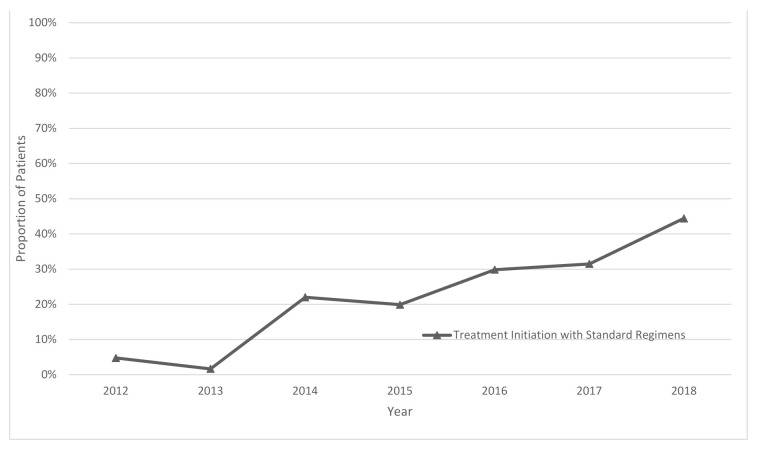
Percentage of patients starting treatment using standardized regimen in accordance with national guidelines for MDR/RR-TB patients registered from 2012 to 2018 in Uzbekistan and assessed under the project of “Evaluation of the TB drug usage for treating MDR TB.

A total of 1481 patients were included in the study. The majority of patients were male (62%), and their mean age was 42 (+/−15) years. During the initial years (2012–2014, hereafter termed the first cohort), 336 patients were included who were mostly from the regions close to Tashkent. After 2014 (hereafter termed the second cohort), the number of patients and the geographical coverage expanded. At the time of data collection, the average treatment duration of the first cohort was 21 months, while in the second cohort, it was 10 months, with only a small proportion of patients having initiated the continuation phase of the treatment. The proportion of patients with laboratory-confirmed MDR/RR-TB was 97% (91% in first and 99% in the second cohort). More details are presented in Table 1, Table 2 and Table 3. The proportion of patients who started the treatment using the standardized regimens in compliance with the national guidelines was 2% in 2012, which increased progressively to 44% in 2018 (Figure 1). Overall, between 2012 and 2018, about 75% of the study population started RR-TB treatment using a non-standardized regimen in contrast to national guidelines (Table 2).

**Figure 2:**
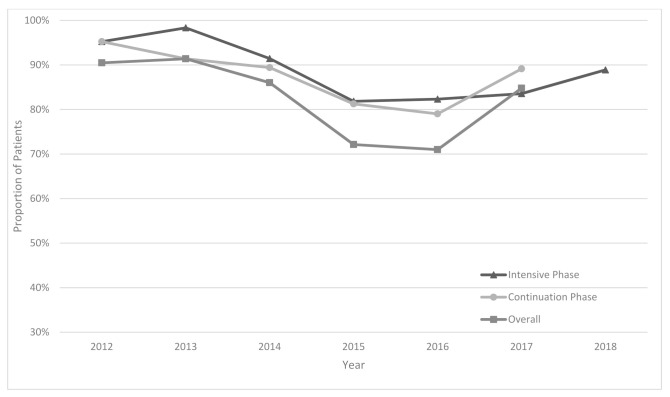
Percentage of patients compliant to weight-based drug dosage among MDR/RR-TB patients registered from 2012 to 2018 in Uzbekistan and assessed under the project of “Evaluation of the TB drug usage for treating MDR TB.

Overall drug dosage compliance ranged from 71% to 91% during the years 2012 to 2017 (Figure 2). In 2017, the compliance was 86%. A slight decrease was observed after 2014. The prolongation of the intensive phase was performed in 42% of patients. The proportion of patients for whom reasons for prolongation were correctly documented in patient cards and in Consilium forms increased from 75% in 2013 to 100% in 2017.

**Figure 3:**
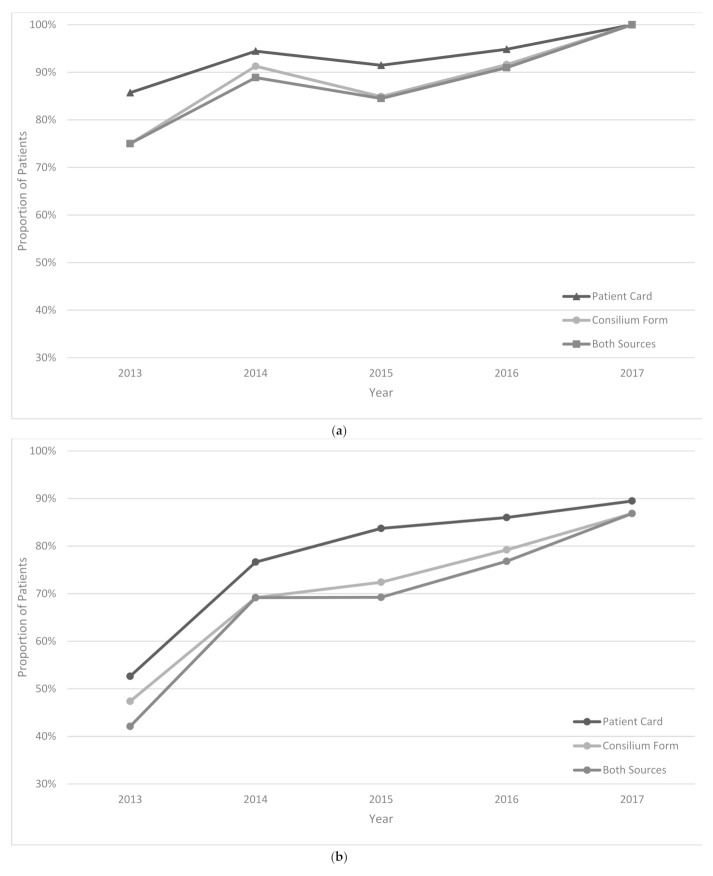

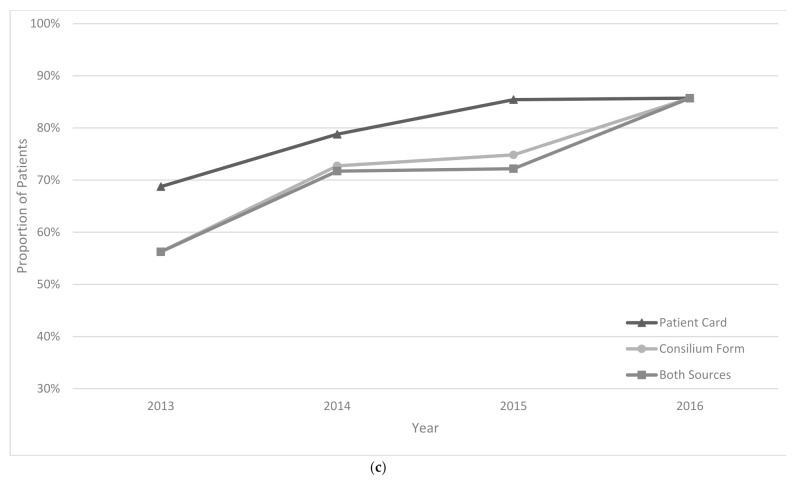
(**a**) Percentage of patients with correct documentation of the reason for intensive phase prolongation among MDR/RR-TB patients registered from 2013 to 2017 in Uzbekistan and assessed under the project of “Evaluation of the TB drug usage for treating MDR TB. (**b**) Percentage with correct documentation of the reason intensive phase treatment change among MDR/RR-TB patients registered from 2013 to 2017 in Uzbekistan and assessed under the project of “Evaluation of the TB drug usage for treating MDR TB. (**c**) Percentage with correct documentation of continuation phase treatment change among MDR/RR-TB patients registered from 2013 to 2017 in Uzbekistan and assessed under the project of “Evaluation of the TB drug usage for treating MDR TB.

The treatment regimen was changed in 44% of patients in the intensive phase and 34% of patients in the continuation phase of treatment. In 2013, treatment changes were correctly documented in 42% and 56% of patients for intensive and continuation phases of treatment, respectively; these increased to 87% and 86% in 2017 (Figure 3a–c).

On adjusted analysis, region, age, sex, regimen, weight at the start of the intensive phase, prolongation of the intensive phase, treatment changes in the intensive and continuation phases were included in the final regression model. Factors that were sig-nificantly associated with compliance with weight-based dosing were region and la-boratory confirmation of resistance. The regions that were identified to be significantly different from Andijon region (reference region—98% compliance) were Farghoana (aRR = 18, 95% CI 4.9–32.6), Qashqadaryo (aRR = 12.9, 95% CI 3.1–31.4), Namangan (aRR = 8.3, 95% CI 1.5–29.5), Republic of Karakalpakstan (aRR = 30.7, 95% CI 17.4–33.8), Surkhondaryo (aRR = 18.7, 95% CI 5.8–32.6), and Sirdaryo (aRR = 10.6, 95% CI 2.4–30.5) regions. In all these regions, the risk of having non-compliance was higher compared to the Andijon region. Additionally, those who did not have laboratory con-firmation of the resistance had a higher risk of non-compliance (aRR = 3.5, 95% CI 1.6–4.7).

## 4. Discussion

This is the first study to review the implementation of MDR/RR-TB treatment regimens in the Republic of Uzbekistan and assess their compliance with the recommendations in the national guidelines. There were three key findings.

First, the usage of the standardized regimens at the treatment initiation was extremely low. In 2012 it was only 2% which started to increase and reached 44% in 2018. This indicates that individualized regimens were used for most patients, which included additional medications. Individualized treatment is only recommended if detailed drug susceptibility test results are available. The initial regimen is expected to be standardized, as most patients are diagnosed with RR-TB and given treatment based on GeneXpert results. Although the use of individualized regimens is permitted by the treatment guidelines and may provide a more effective and tailored treatment to the patients, such a high percentage of individualized regimens may create challenges in procurement and supply chain management, including the potential for stock-outs for some of the drugs. Forecasting the quantities of medicines required for national procurement is based on the use of standardized regimens worldwide [10,11,12]. Thus, such calculations done for Uzbekistan may not be in line with the actual use of drugs and may result in stock-outs. Reasons for the use of individualized regimens were not under the scope of the current study and were not explored. However, possible reasons for the use of non-standardized regimens at the initiation of the treatment might be a history of intolerance to some medications, comorbidities, previous exposure to second-line drugs, and medication stock-outs [4,5,6,13]. Further research is needed to explore the impact of these factors on the use of non-standardized regimens as well as regimen changes during the course of treatment. We found only one similar study that explored DR-TB treatment regimens and dosages in South Africa. There was poor compliance with the national guidelines there as well, with only 30% of patients receiving the correct regimen at the start of the treatment [14].

Second, weight-based dosage compliance during the treatment ranged between 71% and 91%. A slight decrease was observed after 2014, and this may be attributed to the changes in the national guidelines [5]. Thereafter, the compliance increased progressively and reached 85% in 2017 (the latest year where overall compliance was possible to measure). This means that 15% of patients were receiving suboptimal drug dosages with the potential of poor treatment outcomes [7,8]. Higher doses may result in hepatotoxicity [15,16], while lower dosage can have an impact on drug efficacy [17]. Both high and low doses can result in the development of resistance [16]. In a study from South Africa, there was more under-dosing rather than over-dosing ranging from 30% to 40% for different drugs [14]. The analysis revealed that there were two factors associated with non-compliance and the major one was the region. While in many regions, the compliance was as high as 98%, in some, it was significantly lower, reaching 8% in the Republic of Karakalpakstan. The reasons are yet to be explored; however, authors speculate that this may be related to the fact that Médecins Sans Frontières was involved in organizing DR-TB treatment in the Republic of Karakalpakstan, which may be slightly different from that recommended in the national guidelines [18,19,20]. The second factor that was associated with having a higher risk of non-compliance was not having a laboratory confirmation of the resistance. These patients were 3.5 times more likely to receive non-compliant dosage. This may be attributed to the fact that patients who failed the treatment once may receive higher doses of some drugs to increase its efficacy; however, this hypothesis needs to be tested in future research.

Third, 42% of patients had prolongation of the intensive phase, and the treatment was changed in 44% of patients during the intensive phase and 34% of patients during the continuation phase. According to the national guidelines, these changes need to be discussed in medical Consiliums and be properly documented in patient cards and Consilium forms [4,5,6]. The documentation was poor in the early years (42–75%); however, it improved significantly in later years (86–100%). While the practice of documentation is improving, it is still questionable why so many changes are being made to the treatment and why in many patients (in every four out of ten patients), the intensive phase is prolonged.

One of the strengths of this study was the conduct and reporting of the study in line with the Strengthening the Reporting of Observational Studies in Epidemiology (STROBE) guidelines [21]. There was only one study that investigated compliance of RR-TB treatment with the national treatment guidelines in South Africa; thus, this study adds to limited global evidence on this topic. There were several limitations mainly related to the disproportional coverage of the regions in Uzbekistan (in terms of geography and number of patients enrolled), especially in the initial years of the project; thus, comparison between years may be biased, and generalizability of the findings may be limited. Drug susceptibility test results were not collected in the initial project data to allow further exploration of the reasons behind the high proportion of the individualized regimens. The data on TB treatment outcomes were not collected during the initial project, and we did not have access to such data to assess how the compliance may impact outcomes. We did not collect data to explore reasons for starting treatment with an individualized regimen and explain why the proportion of patients with those have decreased over time.

Even with these limitations, there are major programmatic implications resulting from the findings. First, a thorough review of the reasons for the use of individualized, non-standardized regimens is needed throughout the country (more focusing on regions with lower weight-based dosage compliance). This may improve the implementation of the national treatment guidelines and may result in improved patient care and treatment outcomes. Additionally, the country is introducing bedaquiline containing fully-oral short-course treatment regimens, which itself may improve use of the standardized regimens [22].

Second, on-the-job training should be provided (which may be conducted during monitoring visits) to the treating physicians on weight-based dosing to improve proper dosing. Checking the dosages of drugs should be included in the supervision and monitoring checklists. A performance-based payment system for TB doctors (with incentives for those who prescribe the correct dosage for the majority of patients under their care) may help to improve the overall performance of the care system and the dosage practices in particular.

## 5. Conclusions

This study explored compliance with national guidelines in terms of prescribing the standardized treatment regimens, compliance with weight-based drug dosages, and correct documentation of treatment changes among adult MDR/RR-TB patients in Uzbekistan. The majority of patients received non-standardized, individualized treatment regimens at the start of the treatment, and about 15% of patients in the most recent cohort had not received weight-based drug dosages. The documentation of treatment changes has been improving over time, reaching 100% in some years. Further exploration of reasons for non-compliance is needed for improving compliance to guidelines and overall patient care.

## Figures and Tables

**Table 1:** Characteristics of multidrug-resistant (MDR)/rifampicin (RR)-tuberculosis (TB) patients registered from 2012 to 2018 in Uzbekistan and assessed under the project of “Evaluation of the TB drug usage for treating MDR TB”.

Characteristics		Total *n* = 1481	
		*n*, Mean	%, +/−SD ^1^
Regions of the Republic of Uzbekistan	Andijon region	90	6%
	Bukhara region	105	7%
	Jizzakh region	78	5%
	Farghona region	75	5%
	Khorazm region	160	11%
	Qashqadaryo region	180	12%
	Namangan region	78	5%
	Navoi region	145	10%
	Republic of Karakalpakstan	63	4%
	Samarqand region	91	6%
	Surkhondaryo region	84	6%
	Sirdaryo region	89	6%
	Tashkent region	99	7%
	Tashkent city	144	10%
Sex	Female	559	38%
	Male	922	62%
Age		42	+/−15
Weight at treatment start		58	+/−12
Bacteriological Confirmation	Laboratory confirmed	1436	97%
	Clinically diagnosed	45	3%
Treatment outcome	Cured	154	10%
	Treatment completed	124	8%
	Treatment Failure	23	2%
	Died	11	1%
	Lost to Follow-up	22	2%
	Not Evaluated	1147	77%

^1^ Standard deviation.

**Table 2:** Compliance characteristics of MDR/RR-TB patients registered from 2012 to 2018 in Uzbekistan and assessed under the project of “Evaluation of the TB drug usage for treating MDR TB” (Intensive Phase).

Description of Deviations from Treatment Guidelines	Total *n* = 1481	
	*n*	%
Non-standardized regimen at the start of treatment	1113	75%
Intensive Phase Prolonged	627	42%
Treatment Changed	659	44%
Dosage Non-compliant	225	15%

**Table 3:** Compliance characteristics of MDR/RR-TB patients registered from 2012 to 2018 in Uzbekistan and assessed under the project of “Evaluation of the TB drug usage for treating MDR TB” (Continuation Phase).

Description of Deviation from the Treatment Guideline	Total *n* = 919	
	*n*	%
Treatment Changed	308	34%
Dosage Non-compliant	145	16%
Dosage Non-compliant (Overall)	205	22%

## Data Availability

The data that support the findings of this study are available from the corresponding author, (R.U.), upon reasonable request.
